# Laparoscopic intersphincteric resection vs. transanal total mesorectal excision in overweight patients with low rectal cancer

**DOI:** 10.3389/fsurg.2022.984680

**Published:** 2022-10-06

**Authors:** Zhengbiao Li, Qi Wang, Qingbo Feng, Xingqin Wang, Fujian Xu, Ming Xie

**Affiliations:** Department of General Surgery, Digestive Disease Hospital, Affiliated Hospital of Zunyi Medical University, Zunyi, China

**Keywords:** low rectal cancer, TME, laparoscope, intersphincteric resection, transanal total mesorectal excision

## Abstract

**Objective:**

Anus-preserving surgery in overweight patients with low rectal cancer has been a challenge due to the narrow operating space. Intersphincteric resection (ISR) was once a standard therapeutic option for low rectal cancer. The effectiveness of transanal total mesorectal excision (taTME) in treating this group of patients remains uncertain as a new surgical strategy. The aim of this study was to evaluate the short-term effects of taTME with ISR in overweight patients with low rectal cancer.

**Methods:**

A total of 53 patients with low rectal cancer were treated with taTME in 31 cases and ISR in 22 cases. The surgery-related data, pathological manifestations of surgical specimens, postoperative recovery, and postoperative complications were compared.

**Results:**

Patients in both groups completed the surgery successfully. There were no significant differences in operative time, blood loss, anastomotic distance from the anal verge and ileostomy between the two groups (*P* > 0.05). TaTME group performed or virtually finished resection of the rectal mesentery, and no positive cases of Circumferential Resection Margin (CRM) or Distal Resection Margin (DRM) were detected in either group. The number of lymph nodes found in surgical specimens did not change significantly between the two groups (*P* = 0.391). In the subgroup analysis, however, more lymph nodes were detected in female patients undergoing taTME than in male patients (*P* = 0.028). The ISR group took less time to remove the drainage tubes (*P* = 0.013) and the same results were obtained in both groups of male patients in the subgroup analysis (*P* = 0.011). There were no statistically significant differences in time to start liquid diet, time to remove catheters, time to start flatus, time to begin ambulation, postoperative hospital stay, and readmission within 30 days after surgery between the two groups (*P* > 0.05). However, female patients in the taTME group were initiated ambulation earlier than males in the subgroup analysis (*P* = 0.034). The difference was insignificant in the occurrence of postoperative complications between the two groups (*P* > 0.05).

**Conclusion:**

taTME is safe and feasible for the treatment of overweight patients with low rectal cancer.

## Introduction

Colorectal cancer is the third most prevalent kind of malignancies (1). Low rectal malignancies account for 75% of all rectal cancers, which are often classified as the lower rectum within 5 cm of the anal verge (2). Although the treatment of low rectal cancer has evolved extensively in recent decades, surgery remains the key to its care. Total mesorectal excision (TME), proposed by Professor Heald in 1982, is the gold standard for the surgical treatment of rectal cancer (3). Anus-preserving surgery has emerged as the ideal surgical approach pursued assuring tumor removal while maximizing patient life.

Due to low location and restricted operating space, anal preservation for low rectal cancer has been problematic for surgeons. This is especially true for male, obese patients, and narrow pelvis patients (4, 5). Since Sylla (6) et al. reported transanal TME (taTME) in 2010, there has set off a boom in anal preservation surgery for low rectal cancer. TaTME was developed as an alternative technique for mid and low rectal cancer because it could better dissect the presacral plane and the rectoprostatic plane or the rectovaginal plane and better visualize the distal rectum (7). TaTME, due to its bottom-up surgical approach, distinguishes from the traditional surgical route and has a distinct role. Therefore, it has been suggested that taTME surgery may alleviate the surgical challenge encountered by obese, males, and large tumor sizes with low rectal cancer (8). However, there are no worldwide studies to back up this claim.

Laparoscopic-assisted inter sphincteric resection (ISR) is currently one of the most commonly used surgical procedures for the treatment of low rectal cancer in clinical practice. A prospective trial of P. Rouanet showed that the 10-year overall survival (OS) following ISR was 72.2%, and disease-free survival was 60.1% (9), confirming its safety and clinical efficacy.

The purpose of this study was to examine the short-term outcomes of taTME and ISR in the treatment of overweight combined with low rectal cancer, and other complex cases such as male patients, to provide guidance for the clinical treatment of such patients.

## Materials and methods

This study was approved by the Ethics Committee of the Affiliated Hospital of Zunyi Medical University (KLLY-2021-115). All patients signed the informed consent for the surgery. This study was conducted in accordance with the principles of good clinical practice and the Declaration of Helsinki.

### Patient selection

**Figure 1:**
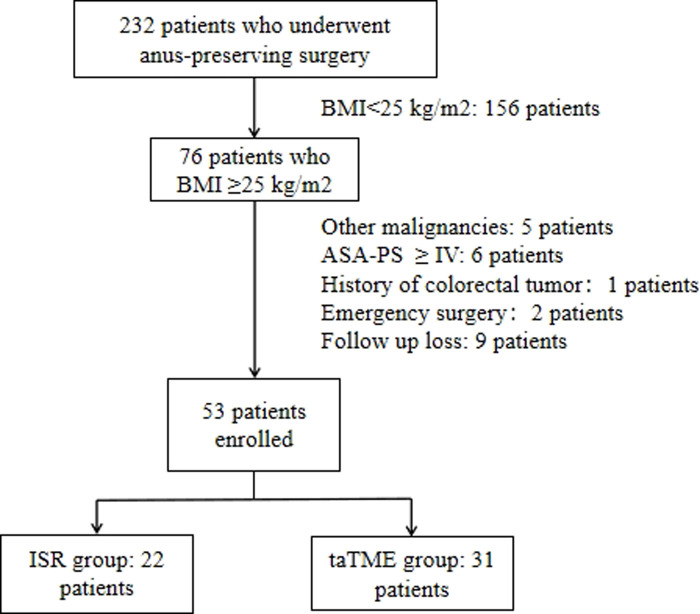
Algorithm for patient selection. Abbreviations: BMI, body mass index; ASA-PS, American Society of Anesthesiologist Physical Status.

Retrospectively analyzed patients with low rectal cancer combined with BMI ≥ 25 kg/m^2^ who underwent taTME or ISR at our hospital from January 2016 to March 2022. Inclusion criteria of patients: (a) rectal adenocarcinoma; (b) distance of the lower margin of the tumor from the anal verge ≤5 cm; (c) cT1-3N0-1MO tumors or downstaging to T1-3N0-1MO after neoadjuvant therapy; (d) resectable tumor on preoperative CT or MRI evaluation. Exclusion criteria: (a) combined with bleeding, bowel obstruction, and perforation requiring emergency surgery; (b) tumor with distant metastasis or a history of colorectal tumor; (c) tumor invading the external anal sphincter, anal levator muscle or involving adjacent organs; (d) American Society of Anesthesiologist Physical Status (ASA-PS) ≥ IV (Figure 1).

### Surgical techniques

**Figure 2:**
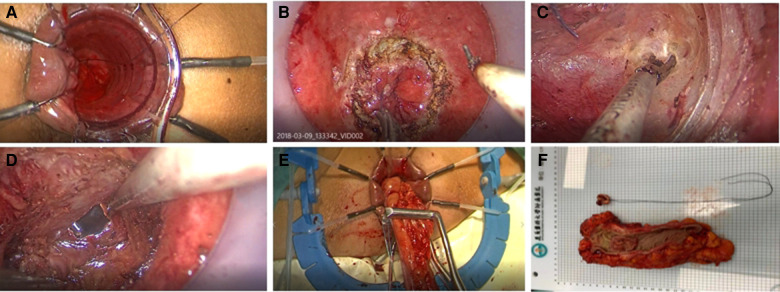
(**A**) Closure of intestinal cavity with purse-string suture (**B**) Layer-by-layer dissection of the entire rectum at a predetermined location (**C**) Bottom-up separation of the rectal mesentery (**D**) Convergence of the transanal and transabdominal groups (**E**) Extraction of the proximal intestine and disconnection of the proximal sigmoid colon (**F**) Pathological specimen.

In taTME group, transanal and transabdominal were performed simultaneously by two groups of surgeons, while the patient in the transabdominal group was operated under the conventional five-hole approach in a lithotomy position. The inferior mesenteric artery root or superior rectal artery root was ligated or resected laparoscopically, and mesenteric lymph nodes were removed. The rectal mesentery was released laparoscopically until it converged with the transanal group according to the TME principle. We will stretch the colonic ligation 15 cm distal to the sacral promontory without tension after the mesangial trimming is complete, If this cannot be achieved, the colon and splenic flexure must be mobilized. For the transanal group, after flushing the intestinal cavity with iodophor water, the intestinal cavity was closed with a purse string at least 1 cm below the tumor. If the tumor is very low and purse-string cannot be performed directly, we can first incise the rectum and free it upward for 1–2 cm before performing a purse-string suture. For patients with rectal cancer whose lower edge of the tumor is above the anorectal ring, the operator directly places a transanal manipulation platform and then completes the taTME operation. If the lower edge of the tumor is located near the anorectal ring, the internal sphincter can be incised first, and the pelvic cavity can be entered by direct visual freeing along the sphincter gap, and then the transanal manipulation platform can be placed when space enough. The rectum was separated from the bottom up until it connected to the transabdominal group before the proximal rectum and sigmoid colon were pulled out of the anus. The sigmoid mesentery and intestinal canal were dissected 12 cm from the proximal end of the tumor, following the specimen removed and a colon-anal canal manual or mechanic anastomosis performed. Depending on the intraoperative situation, the surgeon decided whether to perform further terminal ileostomy. The transanal operation is shown in Figure 2.

**Figure 3:**
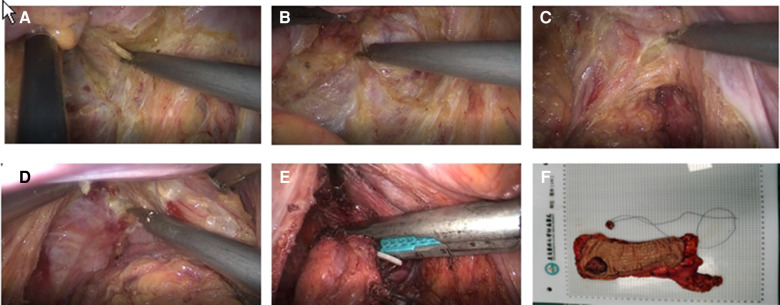
(**A**) Inferior ventral plexus (**B**) Separation of the rectal gap (**C**) Denonvilliers fascia (**D**) Puborectalis muscle and fissure of the anal levator muscle (**E**) Marking the lower edge of the tumor and dissecting the rectum (**F**) Pathological specimen.

In the ISR group, the laparoscopic procedure was the same as taTME group. Depending on the distance between the lower edge of the tumor and the dentate line and the intersphincteric sulcus, the perineal operation was performed as partial ISR, subtotal ISR, or complete ISR, respectively. If the tumor was greater than 2 cm from the dentate line, laparoscopic closure of the rectum could be accomplished by laparoscopic excision of the specimen and removal of the mass through a small left lower abdominal incision. If the tumor was less than 2 cm from the dentate line, the rectum could be dissected through the anus in the intersphincteric sulcus under direct vision. The proximal rectum and sigmoid colon were dragged out *via* the anus, the mesentery was exposed, the sigmoid colon was severed, the specimen was removed, and the colon-anal tube was manually or mechanic anastomosis. According to the intraoperative situation, we decided whether to perform a terminal ileostomy. Figure 3 shows the intraoperative view.

### Observation indicators

The surgery-related data, pathological manifestations of surgical specimens, postoperative recovery, and postoperative complications were compared. Theoretically, women have a wider pelvis, which makes surgical manipulation easier. This has the potential to bias our results. Therefore, we compared the perioperative conditions of men and women in patients undergoing taTME.

### Statistical methods

Statistical analysis was performed using SPSS 25.0. For quantitative data, they were presented as mean ± standard deviation (SD) if they conformed to a normal distribution and analyzed through two independent sample *t*-test methods; otherwise, as median (interquartile range) and analyzed through the Mann–Whitney *U* test. The categorical data were expressed as the number of patients (percentage) and analyzed using the chi-squared test (*χ*2) or Fisher's exact test. *P* < 0.05 was considered a statistically significant difference.

## Results

### Demographic characteristics

A total of 31 patients were included in the taTME group and 22 patients were included in the ISR group. The demographic characteristics of the patients are shown in Table 1. There were no significant variations in body mass index (BMI), gender, age, tumor size, ASA-PS of the patients, distance between tumor and anal verge and preoperative tumor T, N stages between the taTME group and the ISR group (*P* > 0.05).

**Table 1:** Patient characteristics.

Variables	ISR (*n* = 22)	taTME (*n* = 31)	*p*
Age (mean ± SD, years)	60 ± 10.79	55.1 ± 12.28	0.139
Sex (*n*,%)			0.219
Female	5 (22.7%)	12 (38.7%)	
Male	17 (77.3%)	19 (61.3%)	
BMI (mean ± SD, kg/m^2^)	27.671 ± 1.443	27.310 ± 1.550	0.393
Tumor size (mean ± SD, cm)	3.818 ± 0.867	3.548 ± 0.810	0.251
Distance between tumor and anal verg [median (interquartile range), cm]	5 (1)	4 (1.6)	0.054
Neoadjuvant chemoradiation (*n*,%)	1 (4.5%)	2 (6.5%)	1
T stage (*n*,%)			0.465
T_1−2_	12 (54.5%)	20 (64.5%)	
T_3_	10 (45.5%)	11 (35.5%)	
N stage (*n*,%)			0.328
N_0_	15 (68.2%)	17 (54.8%)	
N_1_	7 (31.8%)	14 (45.2%)	
ASA-PS (*n*,%)			0.374
I-II	18 (81.8%)	29 (93.5%)	
III	4 (18.2%)	2 (6.5%)	

### Surgery-related and histopathological results

Patients in both groups successfully completed the operation with no intermediate openings or intraoperative complications. DRM and CRM were negative in the two groups. TaTME group had complete or near-complete resection of the rectal mesentery. The differences in operative time, intraoperative hemorrhage, anastomotic distance from the anus verg, ileostomy ratio, DRM distance and number of lymph nodes detected in the specimen were not statistically significant between the two groups (Table 2).

**Table 2:** Intraoperative and histopathological datas.

Variables	ISR (*n* = 22)	taTME (*n* = 31)	*p*
Operative time (mean ± SD, min)	206.091 ± 52.854	205.645 ± 58.217	0.977
intraoperative hemorrhage [median (interquartile range), ml]	20 (10)	20 (10)	0.953
Anastomotic distance from the anus verg (mean ± SD, cm)	2.273 ± 0.572	2.032 ± 0.741	0.208
Ileostomy (*n*,%)	10 (45.5%)	11 (35.5%0	0.465
DRM distance (mean ± SD, cm)	1.977 ± 0.587	1.739 ± 0.713	0.203
DRM involvement (*n*,%)	0 (%)	0 (%)	–
CRM involvement (*n*,%)	0 (%)	0 (%)	–
Lymph nodes detected (mean ± SD, *n*)	15.000 ± 1.543	14.613 ± 1.647	0.391

### Short-term outcomes after surgery

Postoperative outcomes are shown in Table 3. The differences between the two groups regarding the time of starting the liquid diet, time of catheter removal, time of flatus, time of ambulation, time of postoperative hospital stay, and readmission or complications within 30 days after surgery were insignificant (*P* > 0.05), except for the patients in the ISR group who had earlier catheter drainage tubes (*P* = 0.013).

**Table 3:** Postoperative short-term outcomes.

Variables	ISR (*n* = 22)	taTME (*n* = 31)	*p*
Starting liquid diet (mean ± SD, days)	4.591 ± 1.968	3.677 ± 1.661	0.074
Remove catheter (mean ± SD, days)	4.182 ± 1.680	5.161 ± 2.697	0.138
Remove drainage tubes (mean ± SD, days)	8.091 ± 2.136	10.387 ± 3.792	0.013
Flatus (mean ± SD, days)	3.046 ± 1.430	2.968 ± 1.169	0.829
Ambulation (mean ± SD, days)	6.136 ± 1.983	5.742 ± 1.570	0.423
Postoperative hospital stay (mean ± SD, days)	9.546 ± 2.385	8.742 ± 2.190	0.210
Readmission (*n*,%)	1 (4.5%)	2 (6.5%)	1
Postoperative complication (CD ≥ II) (*n*,%)	3 (13.6%)	7 (22.6%)	0.643
Anastomotic bleeding	0	0	
Urinary disturbance	1	1	
Pneumonia	1	1	
Intestinal necrosis	0	1	
Ileus	1	1	
Pelvic abscess	0	1	
Anastomotic leakage	0	1	
Acute cholecystitis	0	1	

CD, Clavien-Dindo classification.

### Perioperative comparison of male and female patients in taTME group

Female patients had more lymph nodes discovered in their specimens than male patients (*P* = 0.028), and female patients began to ambulate quicker than male patients (*P* = 0.034). There was no statistically significant difference in the remaining perioperative indicators between male and female patients in taTME group (*P* > 0.05) (Table 4).

**Table 4:** Comparison of male and female patients in taTME group.

Variables	Female of taTME (*n* = 12)	Male of taTME (*n* = 19)	*p*
Age (mean ± SD, years)	57.500 ± 11.033	53.579 ± 13.065	0.396
BMI (mean ± SD, kg/m^2^)	27.369 ± 1.693	27.272 ± 1.500	0.868
Tumor size (mean ± SD, cm)	3.250 ± 0.989	3.737 ± 0.632	0.104
Distance between tumor and anal verg (mean ± SD, cm)	3.742 ± 1.014	4.237 ± 0.752	0.130
Operative time (mean ± SD, min)	196.250 ± 49.995	211.579 ± 63.445	0.485
intraoperative hemorrhage (mean ± SD, ml)	16.667 ± 4.924	23.684 ± 14.985	0.129
Anastomotic distance from the anus verg (mean ± SD, cm)	1.917 ± 0.793	2.105 ± 0.714	0.499
Ileostomy (*n*,%)	6 (50%)	5 (26.3%)	0.179
DRM distance (mean ± SD, cm)	1.5 (0.88)	2 (0.80)	0.256
lymph nodes detected (mean ± SD, n)	15.417 ± 0.452	14.105 ± 0.350	0.028
Starting liquid diet (mean ± SD, days)	3.000 ± 0.853	3.895 ± 1.792	0.118
Remove catheter (mean ± SD, days)	5.917 ± 2.678	4.684 ± 2.668	0.221
Remove drainage tubes (mean ± SD, days)	10.000 ± 3.790	10.632 ± 3.876	0.659
Flatus (mean ± SD, days)	3.000 ± 1.279	2.947 ± 1.129	0.905
Ambulation (mean ± SD, days)	5.000 ± 1.044	6.211 ± 1.686	0.034
Postoperative hospital stay (mean ± SD, days)	7.917 ± 2.065	9.263 ± 2.156	0.096
Postoperative complication (CD ≥ II) (*n*,%)	1 (8.3%)	6 (31.6%)	0.201

CD, Clavien-Dindo classification.

### Perioperative comparison between male patients in the ISR and taTME group

The drainage tubes were removed earlier in the ISR group's males (*P* = 0.011). There was no statistically significant difference in the remaining intraoperative and perioperative between the two groups of male patients (*P* > 0.05) (Table 5).

**Table 5:** Comparison between male patients in the ISR and taTME group.

Variables	ISR (*n* = 17)	taTME (*n* = 19)	*p*
Age (mean ± SD, years)	60.294 ± 9.726	53.579 ± 13.065	0.092
BMI (mean ± SD, kg/m^2^)	27.484 ± 1.185	27.272 ± 1.500	0.644
Tumor size (mean ± SD, cm)	3.941 ± 0.933	3.737 ± 0.632	0.443
Distance between tumor and anal verg (mean ± SD, cm)	4.529 ± 0.624	4.237 ± 0.752	0.216
Operative time (mean ± SD, min)	221.118 ± 46.720	211.579 ± 63.445	0.614
intraoperative hemorrhage (mean ± SD, ml)	21.765 ± 14.256	23.684 ± 14.985	0.697
Anastomotic distance from the anus verg (mean ± SD, cm)	2.353 ± 0.493	2.105 ± 0.714	0.242
Ileostomy (*n*,%)	9 (52.9%)	5 (26.3%)	0.102
DRM distance (mean ± SD, cm)	1.941 ± 0.609	1.847 ± 0.164	0.676
lymph nodes detected (mean ± SD, *n*)	14.824 ± 1.629	14.105 ± 1.524	0.181
Starting liquid diet (mean ± SD, days)	4.118 ± 1.833	3.895 ± 1.792	0.715
Remove catheter (mean ± SD, days)	4.294 ± 2.668	4.684 ± 2.668	0.600
Remove drainage tubes (mean ± SD, days)	7.824 ± 1.944	10.632 ± 3.876	0.011
Flatus (mean ± SD, days)	3.059 ± 1.478	2.947 ± 1.129	0.800
Ambulation (mean ± SD, days)	6.059 ± 1.749	6.211 ± 1.686	0.793
Postoperative hospital stay (mean ± SD, days)	9.118 ± 2.342	9.263 ± 2.156	0.847
Postoperative complication (CD ≥ II) (*n*,%)	3 (17.6%)	6 (31.6%)	0.451

CD, Clavien-Dindo classification.

## Discussion

It has been suggested that taTME surgery has potential benefits when applied to male, obese patients with low rectal cancer of large tumor size (8). The surgical safety of taTME in rectal cancer has been confirmed by many studies (10–13), but there are limited studies on its use in overweight patients, therefore it is not clear if there is a significant advantage of performing taTME in this group of patients.

There were no statistically significant differences between the two groups in terms of surgery-related and histopathological results, especially in terms of operative time, intraoperative bleeding and number of lymph nodes detected. Shorter operative time and less intraoperative bleeding may facilitate the patient's postoperative recovery. Some studies have concluded that taTME surgery is faster than transabdominal laparoscopic TME (LapTME) in terms of operative time (14, 15). Theoretically, taTME is performed simultaneously transabdominally and transanally, through a reverse path, from the outside to the inside, and thus should be more rapid in resolving the stenotic space compared to ISR. However, the present study did not confirm this idea. The reasons considered are as follows: (a) Patients with low rectal cancer combined with BMI > 25 kg/m^2^ were selected, in which both surgical approaches face challenges, therefore the differences were not reflected; (b) Both groups included female patients with wider pelvises than men, which reduce the difficulty of surgery and therefore may have an impact on the operative time; (c) Some patients undergoing taTME may be within the learning curve; (d) A lack of sufficient data. When the 2nd reason was considered, subgroup analysis was performed for both groups. However, there were no differences in operative time between male and female patients in the taTME group and in male patients between the ISR and taTME groups. Our study confirmed that taTME did not increase the time to surgery.

DRM and CRM are essential to ensure the quality of TME (16). The quality of surgical resection is strongly associated with the long-term prognosis of the tumor (17) and is recommended for new surgical interventions (18). Obtaining the best quality resection specimen is the most difficult task in the transabdominal approach, especially in obese men with narrow pelvis and large tumors (19, 20). A study based on postoperative magnetic resonance imaging of the pelvis found that residual rectal mesenteric tissue was detected in 3.1% of taTME patients and 46.9% of LapTME patients, suggesting that the integrity of rectal mesenteric resection in taTME patients is significantly better than standard laparoscopic techniques (21). Some studies have suggested that taTME surgery reduces the rate of positive CRM (14, 19, 22, 23), but a recent meta-analysis comparing taTME, ISR, and robotic TME procedures showed that taTME surgery had the worst CRM obtained among these three procedures (24). CRM is considered a more important oncologic indicator than DRM (25–27), and its positivity is considered a strong predictor of local recurrence after rectal cancer surgery (26). However, no positive DRM or CRM was found in this study. In terms of lymph node dissection, the two groups of patients were comparable. However, in subgroup analysis, the number of lymph nodes detected was significantly higher in female than in male in the taTME group. Females usually have wider pelvises than male, suggesting that the narrow space would make the surgical operation more difficult and could affect the quality of surgical resection specimens.

The ISR group had earlier removal of the drainage tubes than the taTME group, and the same results were observed in the subgroup analysis. The placement of postoperative abdominal drains after colorectal surgery can prevent complicated abdominal blood accumulation, reduce the incidence of anastomotic leakage, and facilitate earlier detection of abdominal bleeding, anastomotic leakage or other complications (28). However, it has been suggested that the placement of postoperative abdominal drains may prolong the hospital stay and increase the risk of surgical site infection (29). It took longer for patients in the TaTME group to remove the drainage tubes, which may be explained by the fact that this group was within the learning curve of 50 cases. The operators were less confident and considered it more reliable to leave the drainage tubes for a longer period of time.

Although the rate of postoperative complications was higher in the taTME group, the difference between the two groups were not significant. The complication rate after taTME in previous studies was in the range of 32%–35.7% (14, 15, 22, 30), and this study had lower complications. Urinary disturbance, pneumonia, and Ileus were each found in one case in both groups. Ileus was relieved by conservative treatment. Intestinal necrosis, anastomotic leak, pelvic abscess, and acute cholecystitis were each found in one case in the taTME group. The patient with intestinal necrosis recovered after reoperation and the necrosis was found intraoperatively to be the result of proximal intestinal torsion. Anastomotic leak is one of the most common postoperative complications of rectal cancer (31, 32). The patient with anastomotic leak was relieved by ileostomy. The patient with pelvic abscess was considered to be caused by infection or an undetected occult anastomotic leak, and the patient with acute cholecystitis was considered to be caused by eating a large number of fatty meals after surgery, all of which improved after conservative treatment.

This study still has some limitations: (a) This is a retrospective study and included a small number of cases, which will lead to a large study bias. (b) This study failed to investigate patients' postoperative anal function, long-term quality of life, tumor recurrence rate and patients’ long-term survival rate, so the comparison of the advantages and disadvantages of the two surgical approaches was not adequate. Further results need randomized controlled trials (RCT) with more cases and longer follow-up are needed to evaluate the results present in this study.

## Conclusion

Based on the above findings, taTME is safe and feasible in overweight patients with low rectal cancer. More studies with large samples and high quality are needed to confirm this result.

## Data Availability

The raw data supporting the conclusions of this article will be made available by the authors, without undue reservation.
